# Tetraethyl Orthosilicate-Based Hydrogels for Drug Delivery—Effects of Their Nanoparticulate Structure on Release Properties

**DOI:** 10.3390/gels6040038

**Published:** 2020-10-28

**Authors:** Bogdan A. Serban, Emma Barrett-Catton, Monica A. Serban

**Affiliations:** 1 Department of Biomedical and Pharmaceutical Sciences, University of Montana, Missoula, MT 59812, USA; bogdan.serban@umontana.edu; 2 Department of Bioengineering, Santa Clara University, Santa Clara, CA 95053, USA; ebarrettcatton@scu.edu; 3 Department of Chemistry and Biochemistry, University of Montana, Missoula, MT 59812, USA

**Keywords:** tetraethyl orthosilicate, thixogel, nanoparticles, drug release

## Abstract

Tetraethyl orthosilicate (TEOS)-based hydrogels, with shear stress response and drug releasing properties, can be formulated simply by TEOS hydrolysis followed by volume corrections with aqueous solvents and pH adjustments. Such basic thixotropic hydrogels (thixogels) form via the colloidal aggregation of nanoparticulate silica. Herein, we investigated the effects of the nanoparticulate building blocks on the drug release properties of these materials. Our data indicate that the age of the hydrolyzed TEOS used for the formulation impacts the nanoparticulate structure and stiffness of thixogels. Moreover, the mechanism of formation or the disturbance of the nanoparticulate network significantly affects the release profiles of the incorporated drug. Collectively, our results underline the versatility of these basic, TEOS-only hydrogels for drug delivery applications.

## 1. Introduction

The pharmacological effects of drugs are drastically impacted by their delivery method and overall presentation for absorption and distribution [1,2]. The systemic administration of therapeutics is often associated with adverse effects, and in certain cases, labile therapeutics need to undergo additional costly chemical or dosage form modifications to ensure their effectiveness [3,4]. To address the aforementioned issues, various topical administration alternatives have been explored [5,6,7]. Among these, hydrogels have been particularly useful due to their deployment, engineering, mechanical and biological versatility [8,9,10,11,12,13].

Our group has previously reported the development and characterization of a tetraethyl orthosilicate (TEOS)-based thixotropic drug delivery system for -otic applications [14]. TEOS is an inorganic precursor for the generation of sol–gel transient systems [15], and several of its applications, either as hydrogels [16,17] or as nanoparticle-based drug delivery vehicles, have been reported [18,19]. From a regulatory and commercialization perspective, TEOS-coated super paramagnetic iron oxide nanoparticles (SPION) received FDA approval as a biomedical photoacoustic contrast agent [20]; therefore, TEOS can be a suitable raw material for translational applications.

**Figure 1:**
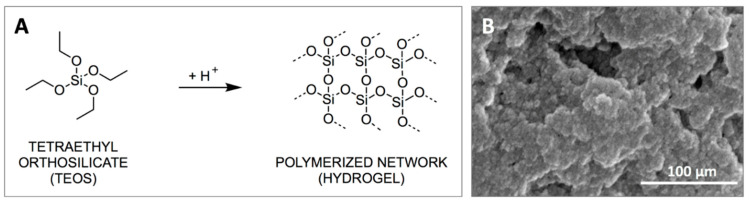
Tetraethyl orthosilicate (TEOS)-based hydrogel formation. (**A**)—reaction mechanism; (**B**)—nanoparticular structure of the hydrogel.

TEOS-based hydrogels form though colloidal aggregation involving nanoparticle assembly dominated by short-range van der Waals forces and surface charge interactions (Figure 1) [21,22]. The nature of these interactions enables the thixotropic properties of these hydrogels, which from a drug delivery system perspective would allow them to be applied as liquids, followed by rapid in situ gelation and drug release [14,22]. We have previously shown that the rheological properties, cytocompatibility and drug release properties of TEOS-based thixogels (TXGs) can be tailored via composition [14,22]. In this work, we focused on understanding the impact of the nanoparticulate hydrogel building blocks and their interactions, on the rheological and importantly, drug release properties of simple TEOS-only hydrogels, in order to enable the rational design of subsequent, application-tailored thixogels. Other research groups have previously reported on the kinetics of silica particle formations from base-driven TEOS hydrolysis and the method-dependent particle size distributions [23,24,25].

## 2. Results

**Figure 2:**
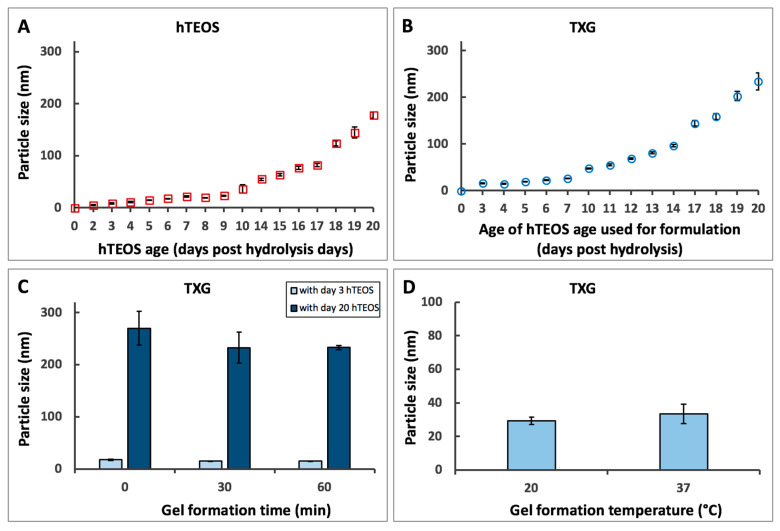
Hydrogel (TXG) formulation effects. (**A**)—time-dependent particle size analysis of hydrolyzed TEOS (hTEOS); (**B**)—particle size analysis of TXG formulated with different age hTEOS; (**C**)—short time effects of hTEOS age on TXG particle size (ANOVA analysis for day 3 hTEOS indicated no statistically significant differences: *p* = 0.076 for alpha = 0.05, F = 4.086 and F crit = 5.143; ANOVA analysis for day 20 hTEOS indicated no statistically significant differences: *p* = 0.989 for alpha = 0.05, F = 0.011 and F crit = 4.786); (**D**)—effects of TXG formation temperature on its particle size (no statistically significant differences, TTEST *p* = 0.3).

Parameters that affect TXG nanoparticle size. As a first step in understanding the parameters affecting the nanoparticulate structure of these TEOS-based hydrogels, we investigated how the size (Z-average of the hydrodynamic diameter) of the hydrolyzed TEOS (hTEOS), which is the precursor for the hydrogels, was impacted by its age, or the time post-activation (Figure 2A). Our data indicate that the size of hTEOS increases exponentially with age, and at 20 days, the recorded hTEOS was around 175 nm compared to ~35 nm at day 10. The age of the hTEOS used to produce the thixogels also impacts the nanoparticle size of the final material (Figure 2B). Specifically, starting with the hTEOS of 8.1 ± 1.5 nm (day 3 hTEOS) would yield thixogels with an initial nanoparticle size of 15.8 ± 1.2 nm. However, starting with hTEOS of 178.3 ± 5.7 nm (day 20 hTEOS) would yield hydrogels of 233.8 ± 18.2 nm. These results would suggest that the thixogels form through the expansion of an initial hTEOS nucleation center, either via self-aggregation, as seen for day 3 TXG, or via the aggregation of smaller nanoparticles onto a larger nucleation center, as seen for the day 20 TXG. Importantly, this result indicates that the nanoparticulate size of the final material can be controlled via the age of the hTEOS.

We then investigated the effect of the duration of the gel formation process on the size of the constituent nanoparticles (Figure 2C). Two separate materials were assessed immediately after thixogel formulation—one was prepared with day 3 hTEOS and the other one with day 20 hTEOS. The day 3 hTEOS used for the formulation of the day 3 TXG was 8.1 ± 1.5 nm in size and the time 0 particle size in the corresponding TXG was 17.1 ± 1.7 nm, while in the time 60 min TXG it was 15.1 ± 0.5 nm. Similarly, the day 20 hTEOS used for the formulation of the corresponding TXG was 178.3 ± 5.7 nm in size and the time 0 particle size in the day 3 TXG was 269.8 ± 32.3 nm, while in the time 60 min TXG it was 232.9 ± 3.6 nm. For both thixogels, regardless of the age of the hTEOS used for formulation, there was no statistically significant difference detected between time 0 versus time 60 min, indicating that the thixogel self-assembly was spontaneous and stable, at least during the monitored timeframe. Interestingly, for both day 3 hTEOS and day 20 hTEOS, respectively, the range of particle size distributions appears to narrow and progress towards monodispersity, potentially indicating that the network assembly reaches an equilibrium between 30 and 60 min of initiation. Based on previous studies on the kinetics of nanoparticle formation [24], the hydrolysis of the first ethoxide is the most likely rate-limiting process of the network formation, followed by the subsequent rapid hydrolysis of the remaining ethoxides and the condensation of Si(OH)_4_, resulting in the formation of monodisperse silica nanoparticles. The same kinetic study indicated that the amount of nanoparticles formed was less than the consumed TEOS [24]. In our methodology, we pre-activated TEOS via the acid hydrolysis prior to treatment with ammonium hydroxide; we therefore believe that the particle size distribution changes observed with our materials were due to a secondary TEOS hydrolysis process driven by the presence of NH_4_OH.

Lastly, we investigated the effect of temperature on the nanoparticulate size of TXG prepared with day 7 hTEOS. The material formulated at 20 °C primarily was comprised of 29.7 ± 2.2 nm particles, while the one formulated at 37 °C comprised of 33.5 ± 5.8 nm particles, with no statistically significant differences noted (Figure 2D).

**Figure 3:**
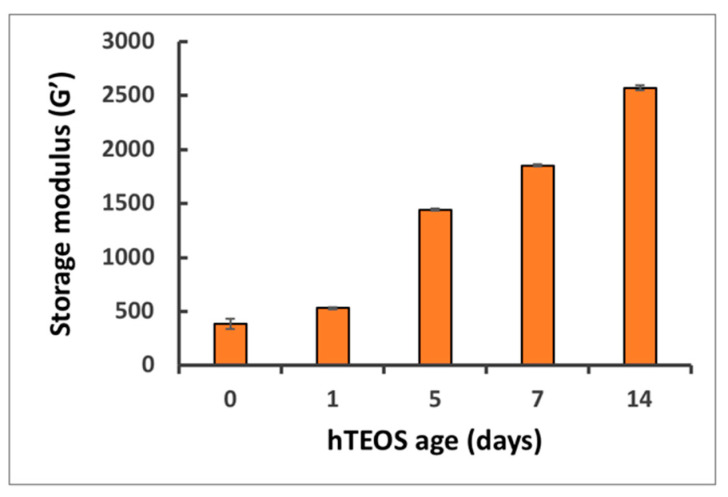
Effects of the age of hTEOS used for the TXG formulation and its stiffness (ANOVA analysis: *p* = 6.39 × 10^−16^ for alpha = 0.05, F = 3909.997 and F crit = 3.478).

Effect of TXG nanoparticulate size on material stiffness. We next sought to understand how the size of the constituent nanoparticles impacts the stiffness of the thixogels. Five different thixogels were evaluated, formulated with day 0 hTEOS, day 1 hTEOS, day 5 hTEOS, day 7 hTEOS and day 14 hTEOS, respectively. The rheological evaluation of their storage moduli (G’) indicated that the size of the hTEOS used for formulation, and intrinsically the size of the thixogel nanoparticulate structure, correlated with the stiffness of the material (Figure 3). Specifically, the starting material with the lowest nanoparticle size yielded the softest material (G’ = 383 Pa) while the hTEOS with the largest nanoparticle size yielded stiffer thixogels (G’ = 2570 Pa). This is an important observation as it provides a simple and effective method to control the final intended thixogel stiffness early on in the manufacturing process, via the starting material used for TXG formulation.

**Figure 4:**
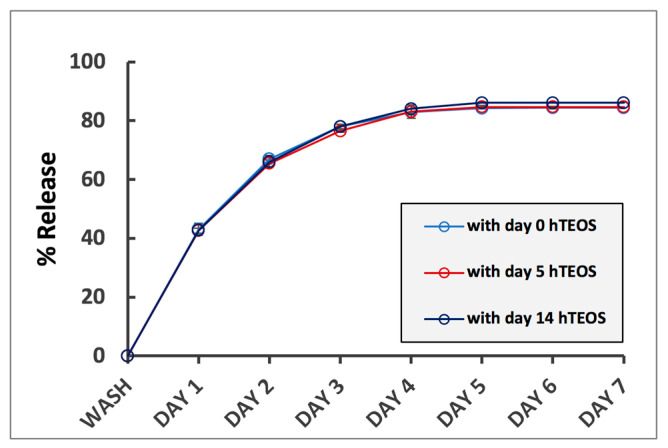
Effect of hTEOS age on the drug release rates from TXG. Error bars are hidden in the plot symbols when not visible.

Effect of TXG nanoparticulate composition on drug release: our group has previously reported that the formulation of the thixogels impacts the rate of release for incorporated drugs [14]. We therefore sought to assess the impact of the TXG nanoparticulate size on the release rates of incorporated drugs. As a drug model, we chose fluorescein, a fluorescent molecule of 332 g/mol that can be easily monitored spectrophotometrically. Thixogels were prepared with day 0 hTEOS, day 5 hTEOS and day 14 hTEOS, respectively, and fluorescein was incorporated into the solutions just prior to the material gelation. As indicated above, the size of the nanoparticles in these three hydrogels were 15.8 ± 1.2 nm, 18.8 ± 0.5 nm and 233.8 ± 18.2 nm, respectively. The release of fluorescein was monitored for 7 days under physiological conditions (37 °C, immersed in phosphate buffered saline). Our results appear to imply that thixogels with smaller particle sizes (made with day 0 and 5 hTEOS) release the model drug at slightly lower rates than the thixogel made with day 14 hTEOS. However, statistical analysis of the data indicated that the drug release rates were similar for the three thixogels, even though the size of their constituent nanoparticles was significantly different (Figure 4). The pH of the thixogels was ~8.5 and based on the pKa values of fluorescein [26], the carboxyl group of the molecule and the xanthene moieties would be deprotonated and the molecule would be dianionic and only able to be retained via weak van der Waals forces with the silica network (most likely counteracted by electrostatic repulsive forces between the two drug delivery system constituents) [27]. Therefore, fluorescein is most likely simply retained in the water aqueous solution entrapped between the constituent nanoparticles.

**Figure 5:**
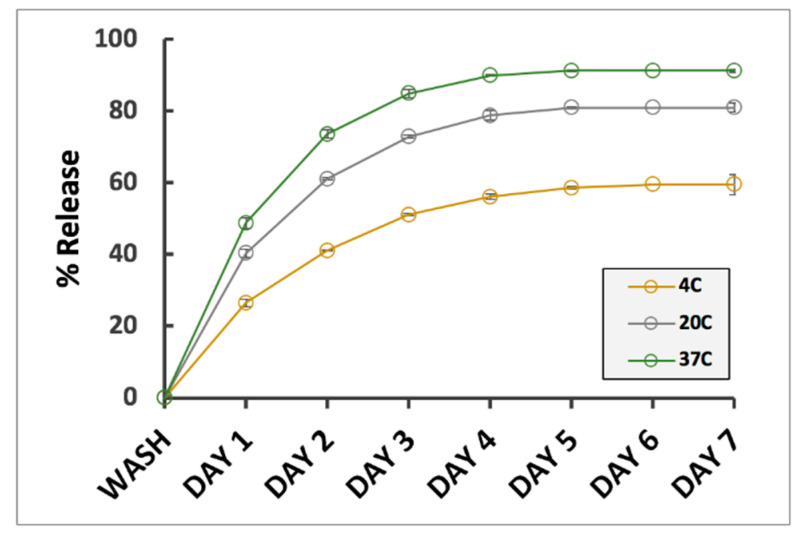
Temperature effect on drug release rates from TXGs. Error bars are hidden in the plot symbols when not visible.

Effect of temperature on drug release rates from TXG. Our previous results indicated that temperature does not significantly alter the nanoparticulate constitution of thixogels (Figure 2D); however, we tried to understand if this parameter impacts the drug release rates from the thixogels. Three different temperatures were selected: 4 °C, corresponding to the typical refrigeration conditions, 20 °C, or room temperature, and 37 °C, reflective of physiological temperature. Our data indicate significant differences in the fluorescein drug release profiles, with faster release occurring at higher temperatures (Figure 5). This finding is in agreement with our previous observation that incorporated drugs are released via diffusion [14]. Moreover, these results appear to indicate that the incorporation of the drug into the thixogels just prior to material gelation results in the distribution of the drug in the aqueous solution entrapped between the constituent nanoparticles.

**Figure 6:**
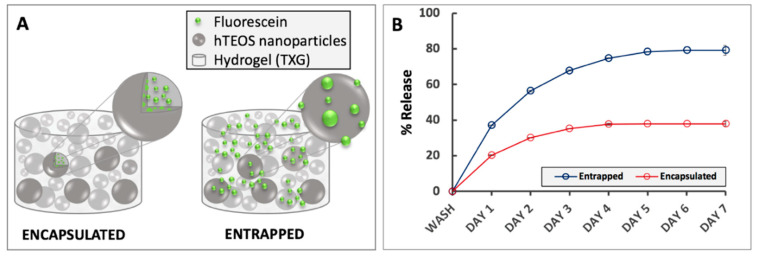
Effects of drug loading strategy on release rates fron TXGs. Error bars are hidden in the plot symbols when not visible. (**A**)—illustration of the drug loading mechanisms into thixogels; (**B**)—release profiles of the encapsulated and encased fluorescein (model drug).

Effects of drug loading mechanism on the release rates from TXG. Based on the above observation and in the context of the thixogel formation mechanism, we sought to understand if the release rates of TXG-incorporated drugs could be controlled at the thixogel formulation level. Specifically, knowing that the thixogels form via the aggregation of hTEOS nanoparticles, we formulated drug-loaded thixogels via two mechanisms. One set was prepared with fluorescein solution, added immediately after hTEOS hydrolysis, which was subsequently allowed to age for 5 days then used for thixogel preparation with encapsulated fluorescein. The second set was prepared ‘traditionally’, by adding the fluorescein solution to day 5 hTEOS just prior to the material gelation with entrapped fluorescein (Figure 6A). Subsequently, we monitored the rates of fluorescein release for 7 days under physiological conditions (Figure 6B).

**Figure 7:**
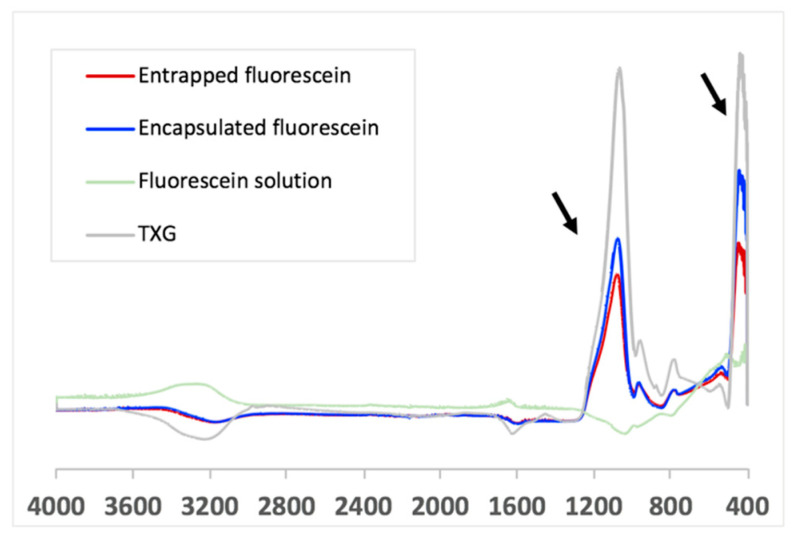
FTIR analysis of the two thixogels, confirming the entrapment and encapsulation of the model drug in the silica nanoparticle network. The arrows indicate the Si–O–Si specific signals (445 cm^−1^, Si–O–Si assymetrical bend; 1088 cm^−1^, Si–O stretch).

Our data indicate that the drug encapsulated in the TXG constituent nanoparticles is released at a significantly lower rate than the drug entrapped in the aqueous solution between the TXG constituent nanoparticles. Our assumption regarding the different fluorescein incorporation into the thixogels via the aforementioned mechanisms was further confirmed by Fourier-transform infrared spectroscopy (FTIR). As postulated based on fluorescein’s chemical structure analysis, the FTIR spectra indicate the simple presence of the drug in the thixogels and the lack any new peaks corresponding to potential physical or chemical interactions between the model drug and silica network (Figure 7). Moreover, in agreement with our proposed drug localization based on the thixogels’ formulation mechanism, the TXG with the aqueously distributed fluorescein shows stronger signals for the model drug molecule (indicated by the arrows), compared to the encapsulated version, in which the Si–O–Si signals are stronger and the fluorescein signal is more shielded [28,29]. Overall, our data confirm the different compartmentalization of the drug in the two formulations, and highlights yet another thixogel property that can be tailored simply through the formulation of the material.

**Figure 8:**
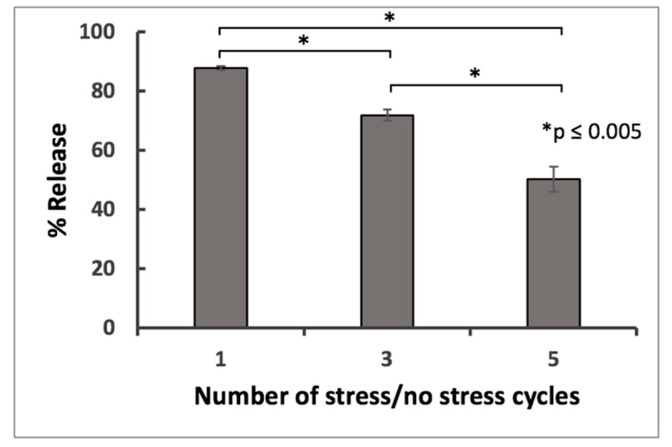
Impact of the number of stress/no stress cycles on the drug release rates from TXGs.

Effect of the stress/no stress cycles on drug release rates from TXG. As we previously reported, the TEOS-based thixogels have the capability to transition between gel/sol states in response to several stress/no stress cycles [22]. We therefore investigated if the number of stress/no stress cycles can impact the amount of drug released from thixogels. For this, we prepared materials with entrapped fluorescein and after gelation, subjected them to one, three and five stress/no stress cycles, respectively. Our results show that subjecting the material to one thixotropic transition results in a burst release of the drug, while multiple thixotropic transitions appear to decrease the amount of drug released (Figure 8). This result indicates that control over drug release properties from thixogels can be achieved even after thixogel formulation, via user-defined thixotropic transitions.

## 3. Discussion

Hydrogels constitute an attractive drug delivery system because of the multiple levels of drug release control, that can help circumvent unwanted pharmacological effects. Our group previously described the development and characterization of a TEOS-based thixotropic drug delivery system for the treatment of otitis externa [14,22]. In this study, we focused on evaluating the levels of control over drug release properties associated with basic TEOS-only thixogel formulations. Additional customizing of thixogel properties can be achieved via the incorporation of supplemental constituents, and those studies have been reported elsewhere [14].

Our results provide a solid understanding of the mechanism of such thixogels formation and its utility in tailoring their drug delivery properties. Moreover, these results can be extrapolated to cost-efficient large scale thixogel manufacturing processes with tailored thixogel parameters and built-in drug release control. Specifically, our data revealed that the age of hTEOS impacts the nanoparticulate structure and stiffness of the final thixogel; this information would dictate the manufacturing parameters of such delivery systems based on their intended biological application and target tissue stiffness [30,31] (i.e., subcutaneous release, intramuscular release). At the manufacturing level, intended drug release rates could be built-in via drug encapsulation versus entrapment, with expected significant pharmacological outcomes. Additionally, the thixogels offer user-level drug release rate control via temperature (corresponding to the anatomical deployment of thixogel, i.e., placement on the skin versus intradermal or subcutaneous injection) and the thixotropic cycling of the materials prior to the deployment to the drug release site. The thixotropic cycling results are intriguing but mechanistically justified. The one cycle results agree with our empirical observations where a small amount of aqueous solution is released from the material as a result of hydrogel reassembly post-stress. Given that the drug is distributed in the aqueous solution between TXG constituent nanoparticles, it is expected that thixotropic cycling would result in a burst release of the drug. However, subsequent thixotropic cycling results in decreased drug release. This is most likely due to the disruption of the initial nanoparticulate structure of the hydrogel, followed by rearrangement and drug encapsulation. For this study, we used fluorescein as a model drug and our data highlighted the interdependence between the molecule’s physicochemical properties and its release pattern, reflective of the nature of interactions with the nanoparticular silica network. These results highlight yet another layer of thixogel-enabled drug delivery control—via the selection of the desired drug structure. Based on our findings, fluorescein-like and non-polar drugs would be released rapidly via diffusion, while positively charged drugs or extensive hydrogen bonds would be expected to be released at slower, more controlled rates. Overall, our work highlights the incredible versatility of these basic, TEOS-only hydrogels for drug delivery applications.

## 4. Conclusions

The results presented herein unveil several design and formulation parameters that could be implemented during the manufacturing process of these materials for tailored drug release properties. In parallel, thixotropic cycling offers an additional layer of user-defined drug release customization. In conjunction with our previous studies on the safety and efficacy of these drug delivery systems, our collective data highlight the exceptional versatility and adaptability of thixotropic TEOS-based hydrogels for drug delivery applications.

## 5. Materials and Methods

Materials: tetraethyl orthosilicate (TEOS) was purchased from Acros Organics (Fair Lawn NJ, USA) and phosphate buffered saline (PBS) was from Corning Inc. (Corning, NY, USA). Fluorescein was purchased from Sigma-Aldrich Chemical Co. (Milwaukee, WI, USA). Acetic acid (HOAc) was from EMD Millipore (Billerica, MA, USA), ammonium hydroxide (NH_4_OH) was from Fisher Chemical (Fair Lawn, NJ, USA).

Analytical Instrumentation: particle size measurements were performed with a Zetasizer Nano (Malvern Analytical, Westborough, MA, USA). Controlled release data were obtained with a FilterMax F5 microplate reader (Molecular Devices, Sunnyvale, CA, USA). Rheological data were acquired with a hybrid Discovery HR-2 Rheometer/Dynamic Mechanical Analyzer (TA Instruments, New Castle, DE, USA).

TEOS hydrolysis and thixogel (TXG) formation: TEOS was hydrolyzed under magnetic stirring at with 0.15 M HOAc for 1.5 h at a 1:9 (*v*/*v*) ratio. Hydrolyzed TEOS (hTEOS) was used immediately or kept/aged under normal environmental conditions for a maximum of 20 days. For TXG preparation, fresh or aged hTEOS was combined with deionized water (diH_2_O) in a 1:1 *v*/*v* ratio. This mix was then vortexed and the pH was adjusted to ~2 with 3.0 N HOAc. After 3 h, the pH was raised to 8.5 with 1.5 N NH_4_OH. Gels formed overnight under normal environmental conditions. Gels were subsequently washed with diH_2_O to remove the residual ethanol (TEOS polymerization reaction by-product).

Drug release studies: fluorescein (MW = 332.31 Da) was used as a hydrophobic drug model because of its ease of monitoring. A fluorescein stock solution of 10 mg/mL was prepared in 0.15 M NH_4_OH. Hydrogel aliquots prior to gelation (1.980 µL) were transferred to 4 mL glass vials containing 20 µL of fluorescein stock solution, to yield a total volume of 2 mL containing 100 µg/mL fluorescein. The mixtures were left overnight at room temperature to form gels with entrapped fluorescein and were then washed with PBS twice to remove ethanol. For drug encapsulation studies, freshly prepared hTEOS was mixed with fluorescein to yield a total volume of 2 mL and a final concentration of 100 µg/mL. The mix along with the hTEOS were kept/aged at room temperature for 5 days. After 5 days, hTEOS (5 day old) was mixed with diH_2_O and processed for gelation induction as described above. The mixtures gelled overnight at room temperature. After washes, 2 mL of PBS were added to each vial. The vials were then placed at 37 °C with no shaking. The release of fluorescein was monitored at 24 h intervals by assaying 100 µL PBS from each vial. The PBS was discarded and replaced with fresh aliquots daily, for each vial. Drug release was monitored by recording the A495 nm values for the supernatants with a FilterMax F5 microplate reader (Molecular Devices, Sunnyvale, CA, USA).

Particle size analyses: to investigate the particulate nature of the thixogels in a hydrated state, 10X diluted TXG aqueous solutions were investigated at 25 °C using a Zetasizer nano ZS Dynamic Light Scattering (DLS) spectrometer (Malvern Instruments Ltd., Malvern, UK). For these experiments, hTEOS, HOAC, NH_4_OH and diH_2_O were passed through a 0.22 µm filter (Merck Millipore Ltd., Tullagreen, Carrigtwohill, Co. Cork, Ireland) then processed for TXG formation as described above. Subsequently, TXG was diluted 10X with filtered diH_2_O. Particle size distributions were determined at different times and temperatures as indicated under Results.

Rheological characterization: all hydrogels were characterized within the material’s pseudo-linear viscoelastic range with a 1.00 mm gap, at 20 °C, unless otherwise specified. Oscillatory strain sweeps for thixotropy investigation were conducted with an 8 mm parallel plate geometry within a strain range of 1–250% and an angular frequency of 10 rad/s. The wait time between the cycles was 30 s. For studies on the effects of gel/sol transitions on drug release thixogels were subjected to 1, 3 or 5 stress/no stress cycles, respectively, then assessed rheologically as described.

Fourier transform infrared spectroscopy (FTIR): the structural conformations of fluorescein-loaded thixogels were analyzed with a Nicolet iS5 FTIR equipped with an iD7 diamond attenuated total reflectance accessory (Thermo Scientific, Waltham, MA, USA). The absorbance of the samples was measured between 4000 and 400 cm^−1^, with 64 scans, and a resolution of 4 cm^−1^. Background spectra of water were collected under the same conditions and subtracted from the samples.

Statistical analysis: Student’s *t*-tests (2-tail, type 3) were employed for 2-group comparisons with α = 0.05. One-way ANOVA was employed for multiple comparisons with single variables with confidence limits of 95% considered significant.
